# Cost-Effectiveness Analysis of Five Systemic Treatments for Unresectable Hepatocellular Carcinoma in China: An Economic Evaluation Based on Network Meta-Analysis

**DOI:** 10.3389/fpubh.2022.869960

**Published:** 2022-04-15

**Authors:** Mingye Zhao, Xingming Pan, Yue Yin, Hongfei Hu, Jifu Wei, Zhaoshi Bai, Wenxi Tang

**Affiliations:** ^1^Department of Pharmacoeconomics, School of International Pharmaceutical Business, China Pharmaceutical University, Nanjing, China; ^2^Center for Pharmacoeconomics and Outcomes Research, China Pharmaceutical University, Nanjing, China; ^3^Jiangsu Institute of Cancer Research, Jiangsu Cancer Hospital, The Affiliated Cancer Hospital of Nanjing Medical University, Nanjing, China

**Keywords:** unresectable hepatocellular carcinoma, partitioned survival, cost-effectiveness analysis, fractional polynomial, network meta-analysis

## Abstract

**Background and Objective:**

Unresectable hepatocellular carcinoma (uHCC) is the main histological subtype of liver cancer and causes a great disease burden in China. We aimed to evaluate the cost-effectiveness of five first-line systemic treatments newly approved in the Chinese market for the treatment of uHCC, namely, sorafenib, lenvatinib, donafenib, sintilimab plus bevacizumab (D + A), and atezolizumab plus bevacizumab (T + A) from the perspective of China's healthcare system, to provide a basis for decision-making.

**Methods:**

We constructed a network meta-analysis of 4 clinical trials and used fractional polynomial models to indirectly compare the effectiveness of treatments. The partitioned survival model was used for cost-effectiveness analysis. Primary model outcomes included the costs in US dollars and health outcomes in quality-adjusted life-years (QALYs) and the incremental cost-effectiveness ratio (ICER) under a willingness-to-pay threshold of $33,521 (3 times the per capita gross domestic product in China) per QALY. We performed deterministic and probabilistic sensitivity analyses to investigate the robustness. To test the effect of active treatment duration on the conclusions, we performed a scenario analysis.

**Results:**

Compared with sorafenib, lenvatinib, donafenib, D + A, and T + A regimens, it yielded an increase of 0.25, 0.30, 0.95, and 1.46 life-years, respectively. Correspondingly, these four therapies yielded an additional 0.16, 0.19, 0.51, and 0.86 QALYs and all four ICERs, $40,667.92/QALY gained, $27,630.63/QALY gained, $51,877.36/QALY gained, and $130,508.44/QALY gained, were higher than $33,521 except for donafenib. T + A was the most effective treatment and donafenib was the most economical option. Sensitivity and scenario analysis results showed that the base-case analysis was highly reliable.

**Conclusion:**

Although combination therapy could greatly improve patients with uHCC survival benefits, under the current WTP, donafenib is still the most economical option.

## Introduction

The 2020 Global Cancer Burden Report released by the WHO International Agency for Research on Cancer stated that liver cancer accounts for 8.3% of cancer-related deaths and is the third leading cause of cancer deaths worldwide (1). Hepatocellular carcinoma (HCC) is the main histological subtype of liver cancer, accounting for approximately 90% of cases of primary hepatic carcinoma (2, 3). Study has shown that the incidence of HCC in China is 35/100,000 population and the burden of disease in China accounts for ~50% of the global burden (4). A survey and analysis of patients with liver cancer in 13 provinces and cities from 2012 to 2014 showed that the average annual direct medical costs for each case were ¥44,850 (5), which represents a major social and economic burden. Although in early stages, the disease can be cured by resection, liver transplantation, or ablation, most patients present with unresectable hepatocellular carcinoma (uHCC) and have a poor prognosis (6–8).

The conventional treatment regimens of uHCC are mainly chemotherapy and radiotherapy (9). Sorafenib is the first molecularly targeted drug to systematically treat uHCC (10), which was approved by the United States Food and Drug Administration (FDA) for the treatment of advanced uHCC in 2007 and it was the sole targeted drug approved by the FDA in the following 10 years. With the subsequent advent of more molecularly targeted drugs, survival in patients with uHCC has been greatly extended. These drugs include those for first-line treatment, such as lenvatinib and donafenib, and drugs for the second-line treatment such as regorafenib, cabozantinib, apatinib, and ramucirumab. The results of analysis for the Chinese population in the REFLECT trial (11, 12) showed that compared with sorafenib, lenvatinib significantly increased patients' overall survival (OS) and progression-free survival (PFS) and increase objective response rate (ORR) by 18%; therefore, it is currently the first choice for increasingly more clinical experts. Chinese subgroup data of the IMbrave150 trial in 2019 (13, 14) showed that the “T + A” regimen [PD-L1 inhibitor atezolizumab (T) combined with the vascular endothelial growth factor (VEGF) inhibitor bevacizumab (A)] increased ORR greatly, and the median OS was more than double that of the sorafenib regimen. Based on the published 14-month data of the phase II/III ORIENT-32 clinical trial (15) in Chinese patients with uHCC, the ORR of sintilimab (D) plus bevacizumab (hereinafter referred to as the “D + A” regimen) was 16% higher than that of the sorafenib regimen, and the OR and PFS rates were 0.65 and 0.53, respectively. The results of the phase II/III ZGDH3 trial (16) investigating donafenib and sorafenib in first-line treatment of advanced HCC in the Chinese population showed that the OS of patients who received the donafenib regimen was significantly higher than the OS of those who received the sorafenib regimen.

The above clinical trial protocols have been approved for liver cancer in China and the control groups are treated with sorafenib. Sorafenib and lenvatinib were approved in 2008 and 2017 and were included in the catalog of medical insurance category B drugs in 2017 and 2021, respectively. Both the D + A and donafenib regimens were approved in 2021 and have been included in the catalog of medical insurance drugs recently. T + A was approved in 2020, but it is the only treatment that has not been covered by medical insurance so far. In the first two quarters of 2021, according to sales data of public hospitals in 20 key Chinese cities, namely, Beijing, Nanjing, and Shanghai, sales (17) of sorafenib, lenvatinib, and atezolizumab totaled ¥124, ¥108, and ¥16 million, respectively; sales data for sintilimab and donafenib are unavailable.

At present, there are no studies on the cost-effectiveness of donafenib and D + A in the treatment of advanced hepatocarcinoma and no studies comparing the cost-effectiveness of T + A, D + A, donafenib, and lenvatinib in pairs or groups. The survival data of the IMbrave150 and RELFECT trials have been updated; furthermore, prices of some drugs have dropped sharply after a new round of healthcare talks. Hence, we used updated Chinese subgroup data and the latest drug prices to re-evaluate the cost-effectiveness of lenvatinib and T + A vs. sorafenib, and drugs for first-line treatment in the above five regimens were compared in groups to provide a basis for decision-making.

## Materials and Methods

### Model Structure

In this study, a partitioned survival model was used to simulate the survival status of patients with uHCC in different periods under various treatments, namely, PFS, progressive disease (PD), and death. The longest simulation period was 10 years, which simulated 97% of the deaths in all groups, about life-long time for advanced liver cancer, and the cycle length was 1 month. Microsoft Excel 2019 was used for model building.

**Figure 1:**
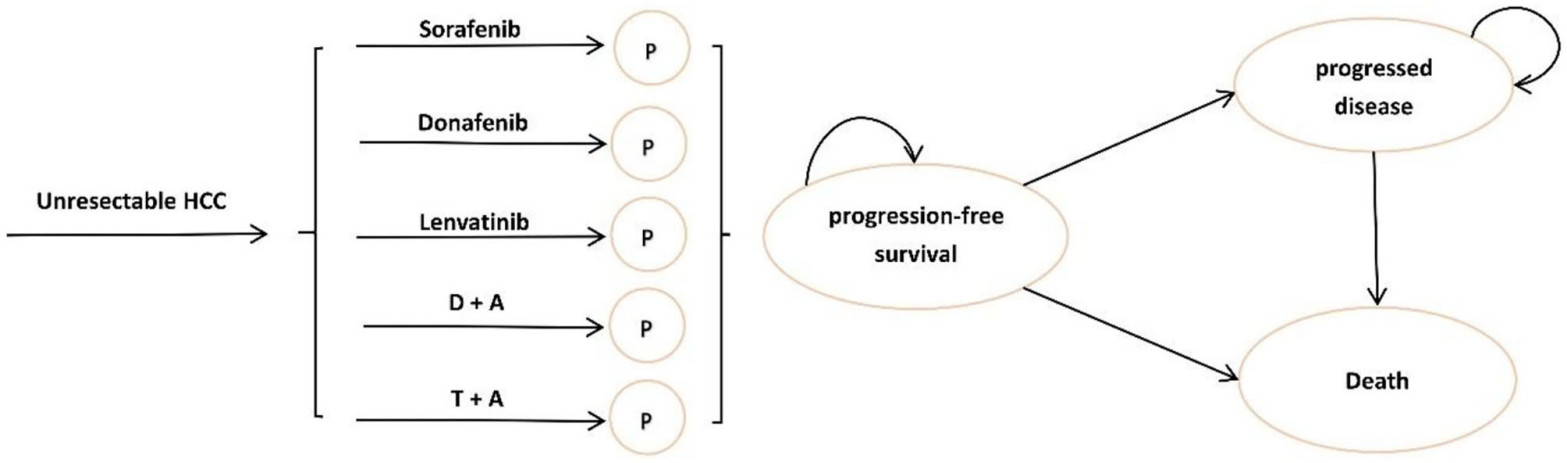
Model structure of a decision tree combining the partitioned survival model. (HCC, hepatocellular carcinoma; P, progression-free survival; D + A, sintilimab plus bevacizumab; T + A, atezolizumab plus bevacizumab).

Our target population was patients with uHCC receiving first-line treatments in China. To determine the most cost-effective first-line systemic treatment regimen for uHCC in this study, we compared five regimens approved in China: (1) sorafenib, (2) lenvatinib, (3) donafenib, (4) atezolizumab plus bevacizumab (T + A), and (5) sintilimab plus bevacizumab (D + A). Figure 1 shows the tree diagram and bubble diagram. Patients would be treated with second-line therapy when their disease progressed, which mainly included tyrosine kinase inhibitor (TKI) therapy (18, 19), immunotherapy (20), and best supportive care (BSC). Furthermore, we assumed that all the patients received BSC 3 months before they died in the base-case analysis. A detailed description of the survival model selection was shown in Supplementary Method in the supplement.

### Clinical Data

We used Chinese subgroup data from the IMbrave150 trial (13), REFLECT trial (11), ORIENT-32 trial (15), and ZGDH3 trial (16) to explore the cost-effectiveness of sorafenib, lenvatinib, T + A, D + A, and donafenib in the treatment of uHCC. The PFS curve of the IMbrave150 Chinese subgroup covered only 16 months of observation and the hazard ratio (HR) of this subgroup was 0.60, which was very close to the HR of the global population (0.59) (14). Therefore, it was assumed that the Chinese subgroup and the treatment group in the total population had the same level of improved PFS relative to the control group; the updated PFS curve of the total population of the IMbrave150 trial was used to replace the PFS curve of the Chinese subgroup. The detailed information of each trial is shown in Supplementary Table 1 in the supplement. The baseline characteristics (namely, age, sex ratio, ethnicity, and indications) of patients in the four trials were basically the same and comparable. The original PFS and OS curves of the four groups are shown in Supplementary Figure 1 in the supplement. The overall quality of the included literature was high, but there was a risk of bias in blinded selection, more details are given in Supplementary Figure 2.

### Model Survival and Progression Estimates

We used GetData Graph Digitizer (version 2.26) to extract survival data from PFS and OS curves. Guyot's method was used to reconstruct individual patient data (21), which is the most accurate data reproduction method currently known for cases in which individual patient data are not available (22). To indirectly compare different regimens and get time-varying HR, we fitted a series of first-order fractional polynomial (FP) models with power parameters −2, −1, −0.5, 0, 0.5, 1, 2, and 3, which included common survival distributions, such as Jansen (23). The calculation formula of time-varying HR is presented in Equations 1, 2, *d*_0_ and *d*_1_ are two key parameters for calculating HR. The log cumulative hazards plots of each trial were used to examine the proportional hazards hypothesis over time. The deviance information criterion (DIC) was used to assess model fit and choose the best model (24, 25). The filtered models were checked by the corresponding survival curves finally. Fixed-effect Bayesian models were used to estimate treatment effects *via* Markov chain Monte Carlo algorithms. Non-informative priors were used to allow the observed trial data to explain effect estimates. We used the R (version 4.1.0), with 3 parallel Markov chains consisting of 100,000 samples after a 10,000 samples burn-in. Finally, we chose the first-order FP model (power parameter = −2) for both OS and PFS, more details are shown in Table 1, the fitted curves are given in Supplementary Figure 4. For PFS, we did not consider the first-order FP model (power parameter = 1) that had smaller DIC as the fitted survival curve violated the clinical reality distinctly judged by clinical experts. Log cumulative hazards plots that showed non-proportional hazards are given in Supplementary Figure 3, OS and PFS curves fitted by all first-order FP models are shown in Supplementary Figure 5. The goodness-of-fit results are shown in Supplementary Table 2 in the supplement. Life-years of all regimens calculated by NMA are given in Table 2.


(1) $$\begin{array}{r} Ln\left(h\left(t\right)\right)=\beta_{0}+\beta_{1}^{*}t^{\text{p}},with\;t^{0}=\log\left(t\right) \end{array}$$

(2) $$\begin{array}{l} Ln\left(HR_{12}\right)=Ln{\left(h\left(t\right)\right)}_{1}-Ln{\left(h\left(t\right)\right)}_{2}\\ \;=\left(\beta_{10}-\beta_{20}\right)+{\left(\beta_{11}-\beta_{21}\right)}^{*}t^{p}=d_{0}+d_{1}^{*}t^{p} \end{array}$$

We derived the expected survival curves for lenvatinib, donafenib, D + A, and T + A by applying the hazard ratios to the reference survival curve. The OS and PFS curves of sorafenib as a reference were derived from the ZGDH3 trail (16), in which OS and PFS curves are the most mature, respectively, the data maturity of OS and PFS was more than 88 and 95%. These data points were then used to fit the following parametric survival functions: Weibull, log-normal, log-logistic, exponential, gamma, and Gompertz models. The eligible survival function was chosen based on the lowest value of the Akaike information criterion (AIC) and visual inspection. The final functions of the sorafenib were log-normal distribution for both the OS and PFS. The log-logistic distribution that had a little lower AIC than the log-normal distribution was judged by clinical experts to have unreasonably fat tails, more details are shown in Table 1 and Supplementary Figure 6 in the supplement. The goodness-of-fit results are shown in Supplementary Table 3.

**Table 1:** Model parameters.

**Item**	**Mean (range)**	**Distribution**	**Sources**
**Clinical input**
Survival model for sorafenib			
Theta for OS	2.40 (2.29–2.50)	Uniform	Lognormal survival model
Sigma for OS	0.95 (0.87–1.04)	Uniform	
Theta for PFS	1.34 (1.25–1.42)	Uniform	
Sigma for PFS	0.77 (0.71–0.84)	uniform	
Parameters for FP model: OS			
d_0_: lenvatinib vs. sorafenib^b^	−0.15 (−0.45–0.15)	Uniform	NMA
d_1_: lenvatinib vs. sorafenib^b^	−1.60 (−3.72–0.02)	Uniform	
d_0_: donafenib vs. sorafenib^b^	−0.18 (−0.37–0.01)	Uniform	
d_1_: donafenib vs. sorafenib^b^	−0.17 (−1.39–0.99)	Uniform	
d_0_: D+A vs. sorafenib^b^	−0.68 (−1.13– −0.22)	Uniform	
d_1_:D+A vs. sorafenib^b^	−0.27 (−2.35–1.78)	Uniform	
d_0_: T+A vs. sorafenib^b^	−0.48 (−0.8– −0.16)	Uniform	
d_1_: T+A vs. sorafenib^b^	−0.23 (−1.59–1.17)	Uniform	
Parameters for FP model:PFS			
d_0_: lenvatinib vs. sorafenib^b^	−0.21 (−0.54–0.13)	Uniform	NMA
d_1_: lenvatinib vs. sorafenib^b^	−0.80 (−1.45– −0.17)	Uniform	
d_0_: donafenib vs. sorafenib^b^	−0.35 (−0.58– −0.12)	Uniform	
d_1_: donafenib vs. sorafenib^b^	0.66 (0.22–1.11)	Uniform	
d_0_: D+A vs. sorafenib^b^	−0.51 (-0.77– −0.24)	Uniform	
d_1_:D+A vs. sorafenib^b^	−0.04 (−0.57–0.50)	Uniform	
d_0_: T+A vs. sorafenib^b^	−0.35 (−0.64– −0.05)	Uniform	
d_1_: T+A vs. sorafenib^b^	−1.63 (−2.26– −1.02)	Uniform	
Regorafenib reduction rate	0.38 (0.36–0.40)	Beta	(26)
Sorafenib reduction rate	0.37 (0.35–0.39)	Beta	(13)
Lenvatinib reduction rate	0.23 (0.22–0.24)	Beta	(11)
Donafenib reduction rate	0.23 (0.22–0.24)	Beta	Assumed
Sorafenib administration frequency	0.90 (0.86–0.95)	Beta	(11)
D+A administration frequency	0.93 (0.88–0.98)	Beta	(15)
Lenvatinib administration frequency	0.92 (0.87–0.96)	Beta	(11)
T+A administration frequency	0.95 (0.90–1.00)	Beta	(13)
Donafenib administration frequency	0.92 (0.87–0.96)	Beta	Assumed
Regorafenib administration frequency	0.90 (0.86–0.95)	Beta	(26)
Tislelizumab administration frequency	0.95 (0.90–1.00)	Beta	Assumed
Probability of grade 1–2 adverse reactions in D+A	0.44 (0.42–0.46)	Beta	(15)
Probability of grade 3 or above adverse reactions in D+A	0.55 (0.52–0.58)	Beta	(15)
Probability of grade 1–2 adverse reactions in sorafenib	0.50 (0.47–0.52)	Beta	(11, 13, 15)
Probability of grade 3 or above adverse reactions in sorafenib	0.67 (0.63–0.70)	Beta	(11, 13, 15)
Probability of grade 1–2 adverse reactions in T+A	0.39 (0.37–0.41)	Beta	(13)
Probability of grade 3 or above adverse reactions in T+A	0.59 (0.56–0.62)	Beta	(13)
Probability of grade 1–2 adverse reactions in lenvatinib	0.34 (0.32–0.36)	Beta	(11)
Probability of grade 3 or above adverse reactions in lenvatinib	0.63 (0.60–0.66)	Beta	(11)
Probability of grade 1–2 adverse reactions in donafenib	0.42 (0.34–0.51)	Beta	(16)
Probability of grade 3 or above adverse reactions in donafenib	0.57 (0.46–0.67)	Beta	(16)
Probability of grade 1–2 adverse reactions in regorafenib	0.33 (0.31–0.35)	Beta	(26)
Probability of grade 3 or above adverse reactions in regorafenib	0.67 (0.64–0.70)	Beta	(26)
Continuing to use the original drug after progression with T+A	0.18 (0.17–0.19)	Beta	(13)
Continuing to use targeted treatment after progression with T+A	0.32 (0.31–0.34)	Beta	(13)
Using Best Support Care after progression with T+A/D+A	0.50 (0.48–0.53)	Beta	(13)
Continuing to use targeted treatment after progression with D+A	0.50 (0.48–0.53)	Beta	Assumed
Continuing to use the original drug after progression with lenvatinib/sorafenib/donafenib	0.03 (0.029–0.032)	Beta	(13)
Continuing to use targeted treatment after progression with lenvatinib/sorafenib/donafenib	0.33 (0.31–0.34)	Beta	(13)
Continuing to use Tislelizumab after progression with lenvatinib/sorafenib/donafenib	0.26 (0.25–0.27)	Beta	(13)
Using Best Support Care after progression with lenvatinib/sorafenib/donafenib	0.38 (0.35–0.41)	Beta	(13)
**Cost ($)**			
Sorafenib per 12,000 mg (Bayer AG, 200 mg, twice a day)	879.11 (703.29–879.11)	Gamma	Local market^a^
Atezolizumab per 1,200 mg (Roche, 1,200 mg, administration once every 3 weeks)	5,058.76 (4,047.01–5,058.76)	Gamma	Local market^a^
Lenvatinib per 120 mg (PATHEONINC, 12 mg/day, body weight≥60 kg; 8 mg/day, body weight <60 kg)	499.71 (399.77–499.71)	Gamma	Local market^a^
Sintilimab per 100 mg (Innovent Biologics, 1,200 mg, administration once every 3 weeks)	166.57 (133.26–166.57)	Gamma	Local market^a^
Donafenib per 4,000 mg (Zelgen Biopharmaceuticals, 200 mg, twice a day)	399.77 (319.82–399.77)	Gamma	Local market^a^
Bevacizumab per 100 mg (T+A group, Roche, 15 mg/kg, administration once every 3 weeks)	231.34 (185.08–231.34)	Gamma	Local market^a^
Bevacizumab per 100 mg (D+A group, Innovent Biologics, 15 mg/kg, administration once every 3 weeks)	176.75(141.40–176.75)	Gamma	Local market^a^
Regorafenib per 1,120 mg (Bayer AG, 160 mg/day, 3 weeks of medications, then discontinuing for 1 week)	744.85 (372.43–744.85)	Gamma	Local market^a^
Tislelizumab per 100 mg (BeiGene, 200 mg intravenously every 3 weeks)	223.63 (178.91–223.63)	Gamma	Local market^a^
Best support care per month	265.08 (212.06–318.10)	Gamma	(27)
Hospice care cost per patient	1,839 (1,519–2,279)	Gamma	(28)
Cost of follow-up and monitoring per month in PFS^c^	114 (86–143)	Gamma	(28)
Cost of follow-up and monitoring per month in PD^c^	210 (157–262)	gamma	(28)
Cost for treatment of adverse reactions of sorafenib	45.6 (36.5–54.8)	Gamma	(11, 13, 15, 18)
Cost for treatment of adverse reactions of D+A	94.2 (75.4–113.1)	Gamma	(15, 18)
Cost for treatment of adverse reactions of T+A	47.0 (37.6–56.4)	Gamma	(13, 18)
Cost for treatment of adverse reactions of lenvatinib	96.5 (77.2–115.8)	Gamma	(11, 18)
Cost for treatment of adverse reactions of donafenib	48.10 (38.48–57.72)	Gamma	(16, 18)
Cost for treatment of adverse reactions of regorafenib	64.3 (51.5–77.2)	Gamma	(18, 26)
**Utilities**			
PFS status utility without adverse reactions	0.76 (0.61–0.91)	Beta	(18, 28, 29)
PD status utility without adverse reactions	0.68 (0.54–0.82)	Beta	(18, 28, 29)
Negative utility of Grade 1–2 adverse reactions	0.01 (0.01–0.02)	Beta	(18, 28, 29)
Negative utility of Grade 3 and above adverse reactions	0.16 (0.11–0.20)	Beta	(18, 28, 29)
**Other**			
Discount	0.05 (0.00–0.08)	Beta	(30)

a *As of December 2021*.

b *HR-related parameter, more details see Equation 2*.

c *Assumed be the same in five treatment groups*.

*D + A, sintilimab plus bevacizumab; T + A, atezolizumab plus bevacizumab; PD, progressed disease; PFS, progression-free survival; AE, adverse effects; FP, fractional polynomial; sd, standard deviation*.

**Table 2:** Results of base-case analysis and scenario analysis.

**Drug**	**Total**			**Only PFS**			**Total**				**Only PFS**			
	**Cost**	**Utility (QALY)**	**Life-years**	**Cost**	**Utility (QALY)**	**Life-years**	**ICER (Sorafenib as a reference standard)**	**ICER (Lenvatinib as a reference standard)**	**ICER (Donafenib as a reference standard)**	**ICER (D+A as a reference standard)**	**ICER (Sorafenib as a reference standard)**	**ICER (Lenvatinib as a reference standard)**	**ICER (Donafenib as a reference standard)**	**ICER (D+A as a reference standard)**
**Base-case analysis**													
Sorafenib	16,614.86	0.91	1.38	4,073.32	0.28	0.39	/	/	/	/	/	/	/	/
Donafenib^a^	21,937.99	1.1	1.68	7,740.16	0.41	0.54	27,630.63	/	/	/	29,735.63	/	/	/
Lenvatinib	23,053.83	1.07	1.63	8,611.27	0.36	0.49	40,667.92	Dominated	/	/	60,084.66	Dominated	/	/
D+A	43,195.21	1.42	2.33	18,312.20	0.42	0.58	51,877.36	66,487.88	56,890.35	/	100,367.32	569,830.35	146,227.70	/
T+A^b^	129,281.72	1.77	2.84	71,551.54	0.49	0.67	130,508.44	160,062.01	150,686.12	245,314.77	330,391.06	788,547.23	489,002.93	853,608.32
**Scenario analysis**													
Sorafenib^a^	19,183.66	0.91	1.38	4,073.32	0.28	0.39	/	/	/	/	/	/	/	/
Donafenib^a^	24,552.34	1.1	1.68	7,740.16	0.41	0.54	27,867.07	/	/	/	29,735.63	/	/	/
Lenvatinib	25,719.93	1.07	1.63	8,611.27	0.36	0.49	41,282.54	Dominated	/	/	60,084.66	Dominated	/	/
D+A	46,355.21	1.42	2.33	18,312.20	0.42	0.58	53,031.48	68,194.54	58,285.35	/	100,367.32	569,830.35	146,226.70	/
T+A^b^	136,163.95	1.77	2.84	71,551.54	0.49	0.67	135,504.93	166,425.92	156,666.76	255,921.76	330,391.06	788,547.23	489,002.93	853,608.32

a *Indicates the best cost-effectiveness (willing to pay = three times per capita gross domestic product)*.

b *Indicates the best clinical effect*.

*PFS, progression-free survival, ICER, incremental cost-effectiveness ratio; QALY, quality-adjusted life-year; D + A, sintilimab plus bevacizumab; T + A, atezolizumab plus bevacizumab*.

### Costs and Utilities

The utility calculated using the EuroQol-5D scale was used to calculate the incremental cost-effectiveness ratio (ICER). The utility of patients with uHCC in PFS and PD states were 0.76 and 0.68, respectively, which were derived from cost-effectiveness analyses considering Chinese patients with uHCC (18, 28); the negative utility of grades 1–2 adverse reactions was 0.01, and grade 3 and above adverse reactions was 0.16 (28, 29).

In this study, from a health system perspective, only the direct costs of disease treatment, namely, drug costs, follow-up cost, monitoring cost, hospice care cost, and costs for treatment of grades 3–4 adverse reactions were considered. In addition, we assumed that the body weight of a patient was 60 kg; medication information is shown in Table 1. Prices for sorafenib, lenvatinib, donafenib, D + A, and T + A were derived from the latest local public bid-winning price (by the end of December 2021). Cost of follow-up and monitoring in PFS or PD were obtained from published literature (28). Specifically, follow-up costs included CT examination, blood test, urinalysis, and blood biochemical examination; costs of monitoring included diagnosis fee, injection fee, nursing fee, and bed fee, more details are given in Table 1.

When calculating costs, the administration frequency, reduction rate, and incidence of adverse drug reactions were considered. The administration frequency of each drug was obtained from the clinical trials, but administration frequency data of tislelizumab in Chinese populations were unavailable. According to the characteristics of its mechanism of action and the occurrence of adverse reactions, we assumed that the administration frequency of tislelizumab was consistent with that of atezolizumab. When an adverse drug reaction occurred, the drug dose would be reduced by half in addition to drug withdrawal. The rates of drug reduction were from the clinical data; the incidences of grade 3 adverse reactions for each drug and the average treatment cost per time are shown in Table 1. Assuming that all the adverse reactions occurred in the first cycle (29) and costs of adverse reactions were derived from literature (18), more details of adverse reaction costs for each drug are available in Supplementary Table 4. Hospice care cost was obtained from a cost-effectiveness analysis in China (28). More details are shown in Table 1. All the costs are expressed in US dollars ($1 = ¥6.4838).

### Cost-Effectiveness Analysis

In this study, cost and utility were discounted and the annual discount rate was 5%, according to *Guidelines for Evaluation of Chinese Pharmacoeconomics* (30). The effectiveness index was life-years and quality-adjusted life-years (QALYs). The ICER and incremental net monetary benefit (INMB) were used to compare the cost-effectiveness of the treatment regimens. According to WHO recommendations, the ICER threshold for this study, or willingness to pay (WTP), was 3 times per capita gross domestic product in China in 2020, namely, $33,521. INMB >0 means economical, the calculation method of INMB is shown in Equation 3.


(3) $$\begin{array}{r} INMB=WTP^{*}\left(E_{2}-E_{1}\right)+\left(C_{2}-C_{1}\right) \end{array}$$

### Sensitivity Analysis and Scenario Analysis

We performed a one-way sensitivity analysis to explore the cost-effectiveness of each regimen when parameters changed between the upper and lower limits and a cyclone graph was plotted to depict the analysis results, INMB was used as a measure of economic efficiency. Monte Carlo simulation was performed for 10,000 iterations and we conducted probabilistic sensitivity analysis (PSA). We used scatter plots and cost-effectiveness acceptability curves (CEACs) to analyze the cost-effectiveness for each regimen with WTP of different values.

In scenario analysis, we considered patients with uHCC would active treatment until death, which was adopted by similar studies (28, 29).

## Results

### Base-Case Analysis Results

After simulation to the endpoint, the cumulative OS time limit, effectiveness, and cost-effectiveness of the five treatment regimens (sorafenib, lenvatinib, donafenib, D + A, and T + A) were obtained, as shown in Table 2. In terms of effectiveness, compared with OS under the sorafenib regimen, patients who received the lenvatinib, donafenib, D + A, and T + A regimens showed an increase of 0.25, 0.30, 0.95, and 1.46 life-years, and a corresponding increase of 0.16, 0.19, 0.51, and 0.86 QALYs. T + A had the best effectiveness both in the OS and PFS states. In terms of cost-effectiveness, for OS, the ICERs of lenvatinib, donafenib, D + A, and T + A compared with sorafenib were $40,667.92/QALY gained, $27,630.63/QALY gained, $51,887.36/QALY gained, and $130,508.44/QALY gained, respectively, all were more than $33,521 except for donafenib, thus donafenib was the most economical regimen for patients with uHCC in China.

### Sensitivity Analysis

#### One-Way Sensitivity Analysis

**Figure 2:**
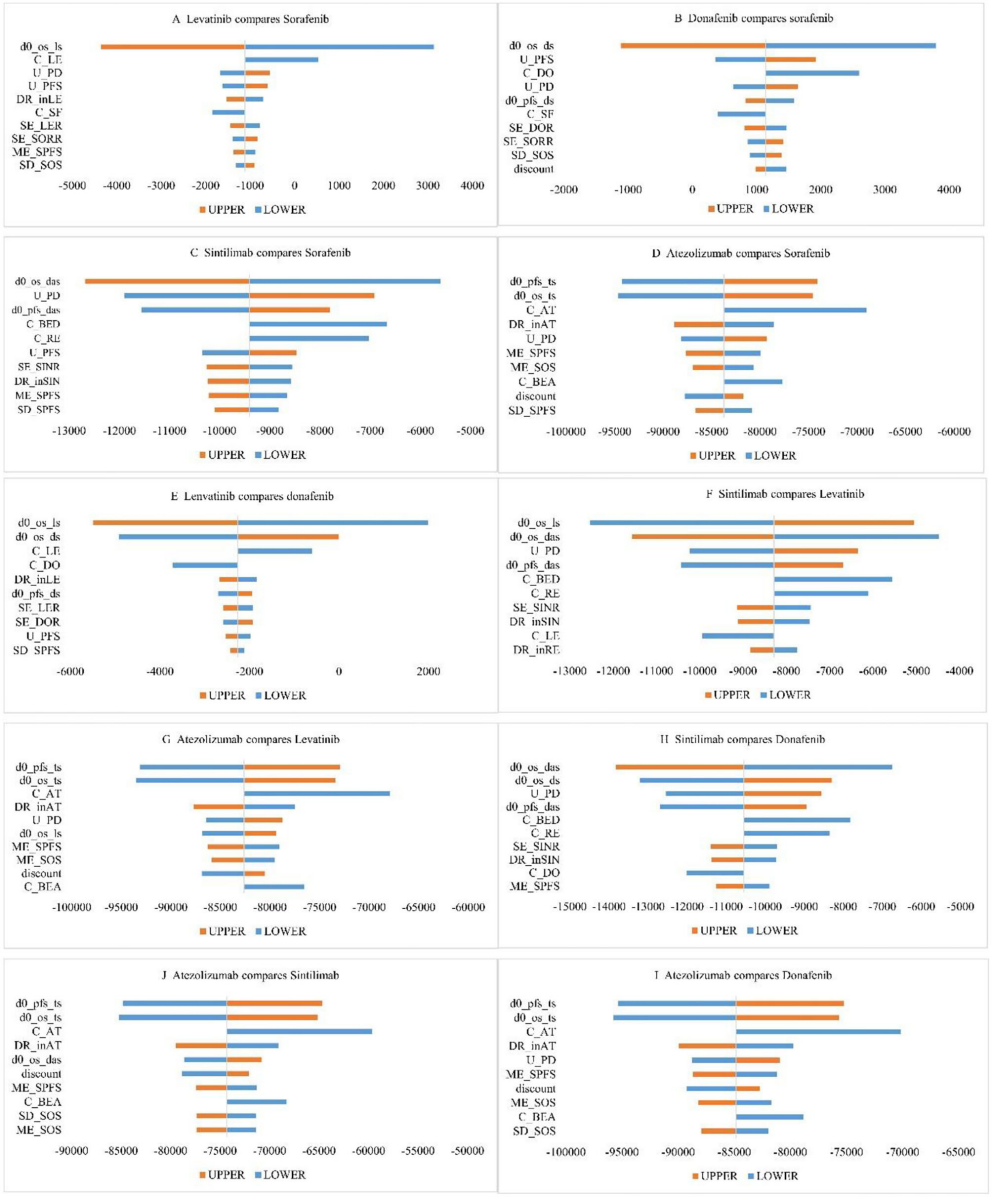
One-way sensitivity analysis chart. (C_AT, unit price of atezolizumab; C_BED, unit price of bevacizumab (D + A group); C_BEA, unit price of bevacizumab (T + A group); C_DO, unit price of donafenib; C_LE, unit price of lenvatinib; C_RE, unit price of regorafenib; C_SF, unit price of sorafenib; DR_inAT, dosage density of T + A; DR_inLE, dosage density of lenvatinib; DR_inSIN, dosage density of D + A; d0_os_das, OS HR (D + A vs sorafenib); d0_os_ds, OS HR (donafenib vs sorafenib);d0_os_ls, OS HR (lenvatinib vs sorafenib); d0_os_ts, OS HR (T + A vs sorafenib); d0_pfs_das, PFS HR (D + A vs sorafenib); d0_pfs_ds, PFS HR (donafenib vs sorafenib); d0_pfs_ts, PFS HR (T + A vs sorafenib); ME_SOS, theta for lognormal model of OS (sorafenib); ME_SPFS, theta for lognormal model of PFS (sorafenib); SD_SOS, sigma for lognormal model of OS (sorafenib); SD_SPFS, sigma for lognormal model of PFS (sorafenib); SE_DOR, probability of TKIs therapy after donafenib progression; SE_LER, probability of TKIs therapy after levatinib progression; SE_SINR, probability of TKIs therapy after D + A progression; SE_SORR, probability of TKIs therapy after sorafenib progression; U_PFS, utility for PFS; U_PD, utility for PD).

Taking $33,521 as the threshold of WTP, we used INMB to measure economic efficiency. Figures 2A–J are the cyclone diagrams of different treatment regimens. As shown in Figure 2, HR-related parameters and utilities for PD and PFS states, drug prices had the greatest impacts on INMB. Cost-effectiveness conclusions of donafenib compared with sorafenib were affected by HR vs. sorafenib; when the price dropped and OS HRs improved, lenvatinib was likely to be cost-effective compared with sorafenib, and lenvatinib had a chance to be the most effective regimen when the OS HRs of lenvatinib and donafenib vs. sorafenib changed. When other parameters fluctuated in the upper and lower limits, the research results were consistent with the base-case analysis, indicating that our base-case analysis results were relatively stable as a whole.

#### Probabilistic Sensitivity Analysis

**Figure 3:**
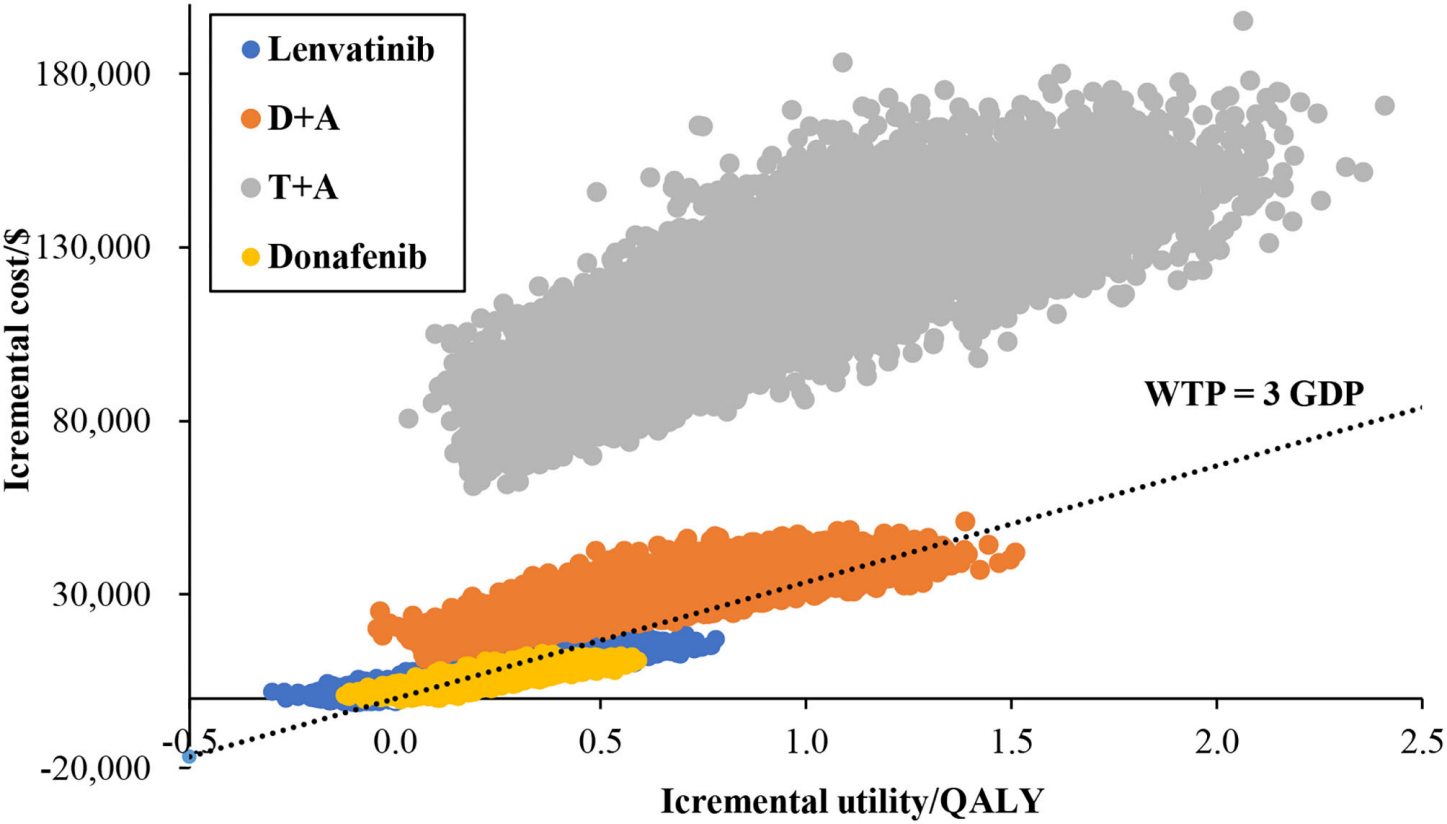
Base-case probabilistic sensitivity analysis: scatter plot (10,000 iterations).

The results of PSA are shown in Figure 3. The results showed that, under the chosen WTP, the probabilities that lenvatinib, donafenib, D + A, and T + A had economic advantages over sorafenib were 31.91, 69.21, 3.44, and 0.00%, respectively.

**Figure 4:**
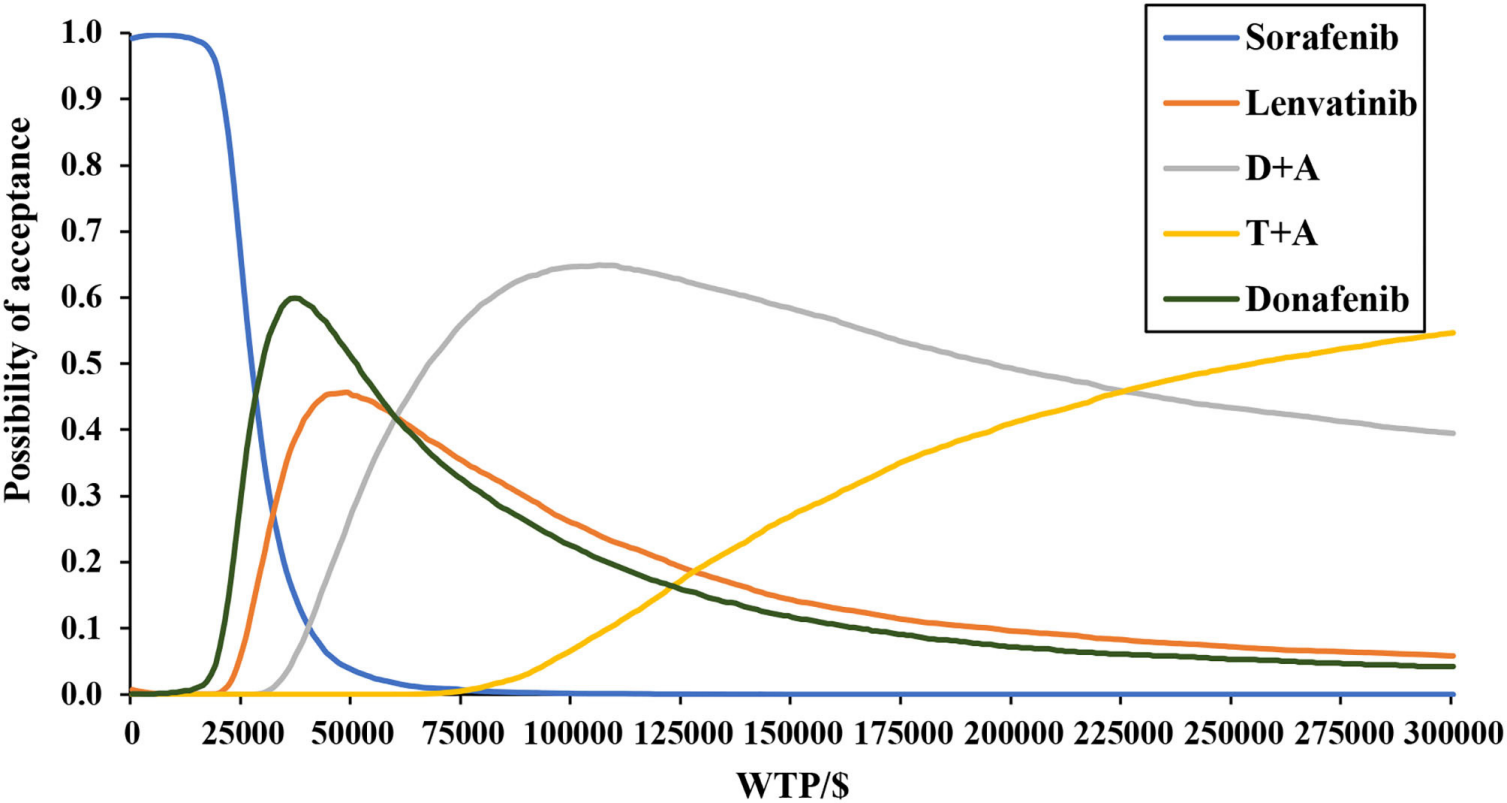
Base-case probabilistic sensitivity analysis: cost-effectiveness acceptability curve (10,000 iterations).

Figure 4 depicts the CEAC, which showed that when using a range of WTP thresholds of $0–27,600/QALY gained, sorafenib was always the most economical option; when WTP was in the range $27,600–66,500, donafenib was the most economical option; when WTP was in the range $66,500–245,300, D + A was the most economical option; and when WTP exceeded $245,300, T + A was the most economical option. Taking the threshold level in China today into account, donafenib was currently the most cost-effective option.

#### Scenario Analysis Results

The results of each scenario analysis are shown in Table 2. Assuming active treatment continued until death, the ICERs of lenvatinib, donafenib, D + A, and T + A compared with sorafenib were $41,282.54/QALY, $27,867.07/QALY, $53,031.48/QALY, and $135,504.93/QALY, respectively. Overall, the results of scenario analysis were consistent with the conclusions of the base-case analysis, verifying the robustness of the conclusions of the base-case analysis. The scatter plot and CEAC are given in Supplementary Figure 7 in the supplement.

## Discussion

In this study, we explored the cost and effect of sorafenib, lenvatinib, donafenib, D + A, and T + A in the treatment of uHCC. The final result showed that the T + A regimen was the most effective and the ranking of cost-effectiveness was as follows: donafenib > sorafenib > lenvatinib > D + A > T + A. Both the deterministic sensitivity analysis and PSA proved the robustness of the results. The scenario analysis showed that active treatment duration would not affect the conclusion.

To date, several articles have evaluated the cost-effectiveness of lenvatinib and sorafenib and T+A and sorafenib in the treatment of patients with uHCC in China. Wen et al. (18) and Hou and Wu (28) evaluated the cost-effectiveness of T + A and sorafenib from the perspective of the healthcare system in China and the conclusions were consistent with those of this study. Cai et al. (31) confirmed that lenvatinib was economical compared to sorafenib when considered donations. Relevant literature outside of China (29, 32–34) showed that ICERs of lenvatinib and T + A compared with sorafenib were significantly higher than the threshold in China, which indicated that lenvatinib and T + A were not more cost-effective than sorafenib in China.

Donafenib has listed in 2021 and was included in the latest medical insurance list. The ZGDH3 trial (16) showed that donafenib improved OS and PFS survival compared with sorafenib, and the price of donafenib dropped by 69% recently, so donafenib was economical compared to other targeted drugs, namely, sorafenib and lenvatinib. Immunosuppressive agents tend to be more expensive, such as atezolizumab and sintilimab combined with VEGF inhibitor. Furthermore, while these drugs prolonged survival (13, 15), they also caused a great economic burden of disease, which may be another reason why combined therapies were not economical. Given that the threshold level will not change much in the next few years, assuming that it remains unchanged, it is expected that the price of D + A drops by 64% and the price of T + A drops by 81%, which will be more cost-effective than donafenib at the current price level.

With no direct randomized controlled trials between groups of drugs, indirect comparisons are necessary. Most previous studies (18, 29, 32–38) have used a common control drug as a bridge and adopted the constant HR assumption. This method requires that the KM curves of the test group and control group obey the assumption of equal proportions. However, the survival curves of drugs (11, 13, 15, 16, 39–43) do not obey the above assumptions usually. Jansen et al. (23) developed fractional polynomials based on non-proportional hazards, and (network) meta-analysis of survival data with models where the treatment effect is represented with several parameters using fractional polynomials can be more closely fitted to the available data than meta-analysis based on the constant hazard ratio. The 4 trials included in this study were all verified to be non-proportional hazards ratios; hence, the FP model based on non-proportional hazards was used.

When the disease progresses, patients may choose a variety of second-line treatments, and the survival time in the PD state is not uniform, which makes the calculation of the treatment cost of PD status very difficult. Similar economic evaluation studies (28, 29) directly chose the average cost of second-line treatment from other research, which ignored the heterogeneity of patients in different studies and also did not reflect the target patients' survival status in PD state well. In our studies, we carefully considered the patient's subsequent treatment options and calculated the cost during PD state based on the patient's selected treatment options and survival status.

To the best of our knowledge, it is the first cost-effectiveness analysis of donafenib and D + A in the treatment of uHCC, and the efficacy and cost-effectiveness of first-line treatment of uHCC approved in China were compared in groups for the first time. This study is important for patients, clinicians, and payers, given the uncertainty about the optimal treatment for uHCC, which causes serious morbidity and mortality in China. Furthermore, our cost-effectiveness analysis can inform value-based decision-making for health systems. In addition, we closely modeled the observed the Kaplan–Meier curves and constructed a network meta-analysis based on the FP model with which time-varying HRs were calculated. This analysis is necessary given that non-proportional hazards were detected in the chosen trials, which has not been addressed by previous reviews (35–38).

However, owing to the lack of direct comparisons of survival data among drugs, uncertainty remains in the results. In addition, owing to a lack of individual data, we assumed that bodyweight is 60 kg and that adverse reactions occur in the first cycle, which affects the calculation of the cost and utility to a certain extent. Regarding the choice of treatment regimens after disease progression, there is no real-world evidence, so the best hypothesis was put forth according to actual clinical applications. Finally, costs and utilities came from different groups, contributing to the bias of results to some extent.

## Conclusion

In this study, we showed that the effectiveness during the OS period was ranked as follows: T + A > D + A > donafenib > lenvatinib > sorafenib and the ranking of cost-effectiveness was as follows: donafenib > sorafenib > lenvatinib > D + A > T + A. Although combination therapies (D + A and T + A) have greatly improved the survival benefit of patients, donafenib is still the most economical option for patients with uHCC due to its low price. It is expected that these regimens may be more widely adopted when the price of these drugs drops and the WTP threshold increases in the future.

## Data Availability Statement

The original contributions presented in the study are included in the article/Supplementary Material, further inquiries can be directed to the corresponding author.

## Ethics Statement

Ethical review and approval was not required for the study on human participants in accordance with the local legislation and institutional requirements. Written informed consent for participation was not required for this study in accordance with the national legislation and the institutional requirements. Written informed consent was not obtained from the individual(s) for the publication of any potentially identifiable images or data included in this article.

## Author Contributions

The conception and design of this study were primarily conducted by WT. The drafting of the article was mainly the responsibility of MZ and XP. All authors have reviewed the analysis, interpretation of the data, contributed to the drafting of the manuscript, revising the manuscript for important intellectual content, approved the final version to be published, and agree to be accountable for all the aspects of this study.

## Funding

This study was supported by the General Program of the National Natural Science Foundation of China (72174207), the Key Projects of the National Natural Science Foundation of China (71734003), and the National Natural Science Foundation of China Youth Project (71603278).

## Conflict of Interest

The authors declare that the research was conducted in the absence of any commercial or financial relationships that could be construed as a potential conflict of interest.

## Publisher's Note

All claims expressed in this article are solely those of the authors and do not necessarily represent those of their affiliated organizations, or those of the publisher, the editors and the reviewers. Any product that may be evaluated in this article, or claim that may be made by its manufacturer, is not guaranteed or endorsed by the publisher.
