# Supplementary material for: Cost-Effectiveness Analysis of Five Systemic Treatments for Unresectable Hepatocellular Carcinoma in China: An Economic Evaluation Based on Network Meta-Analysis
Source: Front Public Health. 2022 Apr 15;10:869960. doi: 10.3389/fpubh.2022.869960 (PMC9051228; doi:10.3389/fpubh.2022.869960)
Supplement: Supplementary file 1 [file Data_Sheet_1.DOCX]

**Supplementary Materials and Methods**

Cost-Effectiveness Analysis of Five Systemic Treatments for Unresectable Hepatocellular Carcinoma in China: An Economic Evaluation Based on Network Meta-Analysis

**Catlogs**

[eTable 1 Information of REFLECT trial, IMbrave150 trial, ORIENT-32 trail, and ZGDH3 Trail 1](#_Toc97565565)

[eFigure 1 Original PFS and OS Curves of IMbrave150 Chinese subgroup, REFLECT trial Chinese subgroup, ORIENT-32 trial, and ZGDH3 trial 3](#_Toc97565566)

[(PFS, progression-free survival; OS, overall survival) 3](#_Toc97565567)

[eFigure 2 Summary of Results from Assess ment of Studies Using the Cochrane Risk of Bias Tool 4](#_Toc97565568)

[eFigure 3 Log Cumulative Hazards Ratios Plots for OS and PFS 5](#_Toc97565569)

[(D+A, sintilimab plus bevacizumab; T+A, atezolizumab plus bevacizumab) 5](#_Toc97565570)

[eFigure 4 OS and PFS Curves for the Best Fitted Fractional Polynomial Models 6](#_Toc97565571)

[(D+A, sintilimab plus bevacizumab; T+A, atezolizumab plus bevacizumab) 6](#_Toc97565572)

[eTbale 2 The Goodness-of-Fit Results for Fractional Polynomials models 7](#_Toc97565573)

[eTable 3 The Goodness-of-Fit Results for Different Disturbutions for Sorafenib Curves 8](#_Toc97565574)

[eTable 4 Adverse Reactions of Each Drug 8](#_Toc97565575)

[eFigure 6 OS and PFS Curves Fitted by All Distributions 10](#_Toc97565576)

[(KM, Kaplan-Meier) 10](#_Toc97565577)

[eFigure 7 Probabilistic Sensitivity Analysis for Scenario Analysis: Scatter Plot (A) and Cost-Effectiveness Acceptability Curve (B, 10,000 iterations) 11](#_Toc97565578)

[(D+A, sintilimab plus bevacizumab; T+A, atezolizumab plus bevacizumab) 11](#_Toc97565579)

[eMethod 12](#_Toc97565580)

# eTable 1 Information of REFLECT trial, IMbrave150 trial, ORIENT-32 trail, and ZGDH3 Trail

| Trial title | Group | Indications | Patient source | Medications and therapies | | Sample size | Average age | Proportion of males (%) | ECOG PS | MVI or EHS | Child-pugh classification standard | BCLC stage | Baseline AFP level (median, ng/mL) | HR(PFS) | HR(OS) | Median PFS (month) | Median OS (month) | Objective response rate |
| --- | --- | --- | --- | --- | --- | --- | --- | --- | --- | --- | --- | --- | --- | --- | --- | --- | --- | --- |
| REFLECT Trial Chinese Subgroup | Treatment group | Advanced uHCC | China | Lenvatinib | | 144 | 55.6 | 88.19 | 1:56% | 66% | A:100% | B: 15%; C: 85% | 267.1(>200ng/mL: 55%) | 0.55  (0.42-0.72) | 0.73  (0.55-0.96) | 7.4  (6.9–8.8) | 13.6  (12.1–14.9) | 22% |
|  | Control group |  |  | Sorafenib | | 144 | 57.2 | 81.25 | 1:54% | 63% | A:100% | B: 17%; C: 83% | 72.7  (>200ng/mL: 40%) |  |  | 3.7  (3.6–4.6) | 12.3  (10.4–13.9) | 8% |
| IMbrave 150 Chinese subgroup | Treatment group | Advanced uHCC | China | Atezolizumab + Bevacizumab | | 133 | 57 | 87 | 1:41% | 83% | A:99% | B: 11%; C: 86% | >400ng/mL: 44% | 0.65  (0.53-0.81) | 0.53  (0.35-0.80) | 6.9  (5.7–8.6) | 24.0  (17.1–NR) | 25% |
|  | Control group |  |  | Sorafenib | | 61 | 60 | 80% | 1:49% | 87% | A:100% | B: 5%;  C: 93% | >400ng/mL: 49% |  |  | 4.3  (4.0–5.6) | 11.4  (6.7–16.1) | 7% |
| ORIENT-32 | Treatment group | Advanced uHCC | China | Sintilimab +Bevacizumab | | 380 | 53 | 88% | 1:52% | 80% | A:96% | B: 15%; C: 85% | >400ng/mL: 43%) | 0.56  (0.46–0.70) | 0.57  (0.43–0.75) | 4.6  (4.1–5.7) | NR | 21% |
|  | Control group |  |  | Sorafenib | | 191 | 54 | 90% | 1:52% | 79% | A:95% | B: 14%; C: 86% | >400ng/mL: 42% |  |  | 2.8  (2.7–3.2) | 13.2 (10.4–NR) | 4% |
| ZGDH3 | Treatment group | Advanced uHCC | China | Donafenib | 328 | | 53 | 86% | 1:61% | 73% | A:99% | B: 13% | ≥400ng/mL: 53% | 0.909  (0.763-1.082) | 0.831  (0.699-0.988) | 3.7  (3.0-3.7) | 12.1  (10.3-13.4) | 5% |
|  | Control group |  |  | Sorafenib | 331 | | 53 | 88% | 1:67% | 73% | A:96% | B: 12% | ≥400ng/mL: 53% |  |  | 3.6  (2.4-3.7) | 10.3  (9.2-12.0) | 3% |

Abbreviations: uHCC, unresectable hepatocellular carcinoma; NR, not reported; ECOG PS, Eastern Cooperative Oncology Group Performance Status; MVI, macrovascular invasion EHS, extrahepatic spread; BCLC, Barcelona Clinic Liver Cancer; AFP, alpha-fetoprotein HR, hazard ratio; OS, overall survival; PFS, progression-free survival.


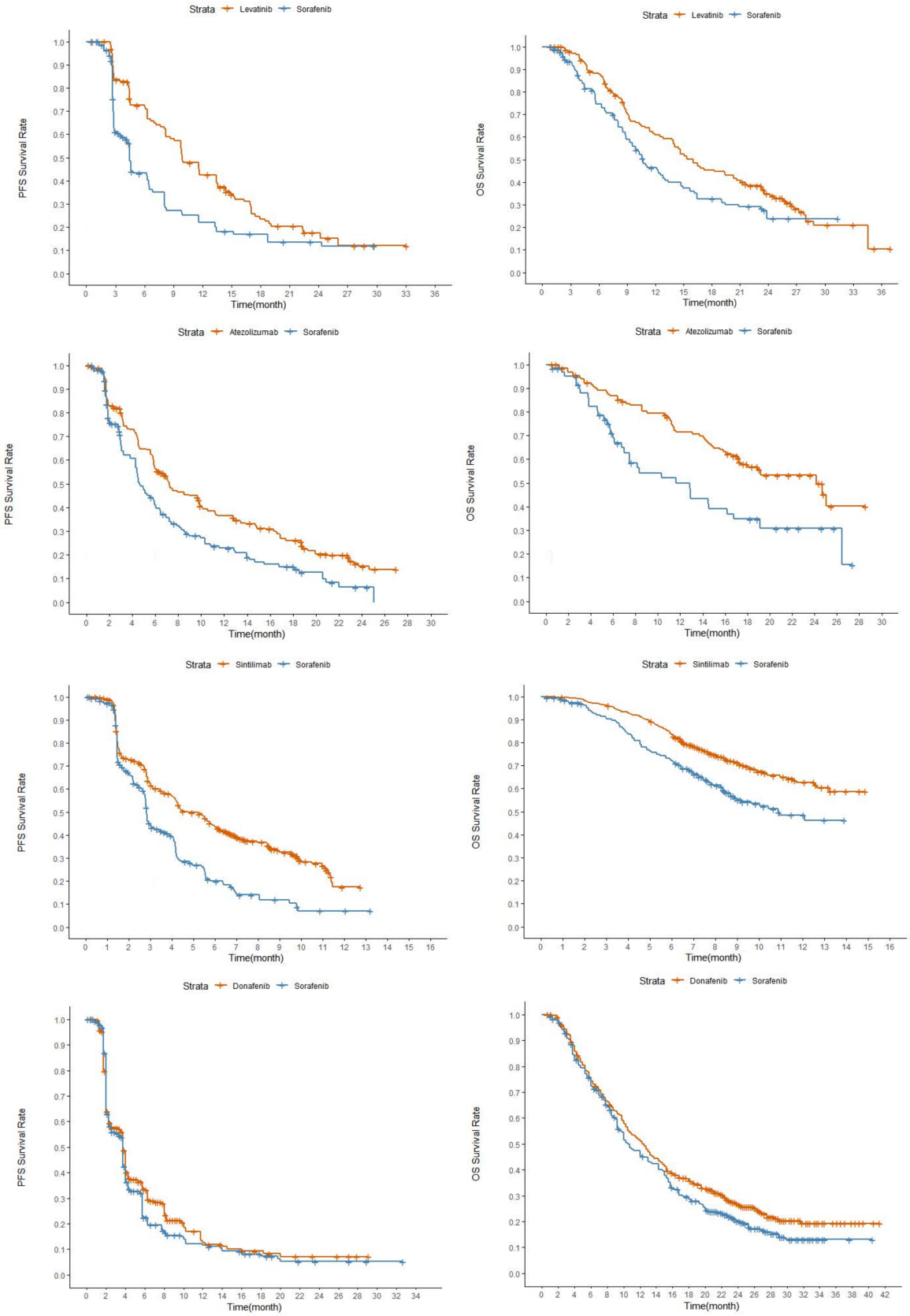


# eFigure 1 Original PFS and OS Curves of IMbrave150 Chinese subgroup, REFLECT trial Chinese subgroup, ORIENT-32 trial, and ZGDH3 trial

# (PFS, progression-free survival; OS, overall survival)


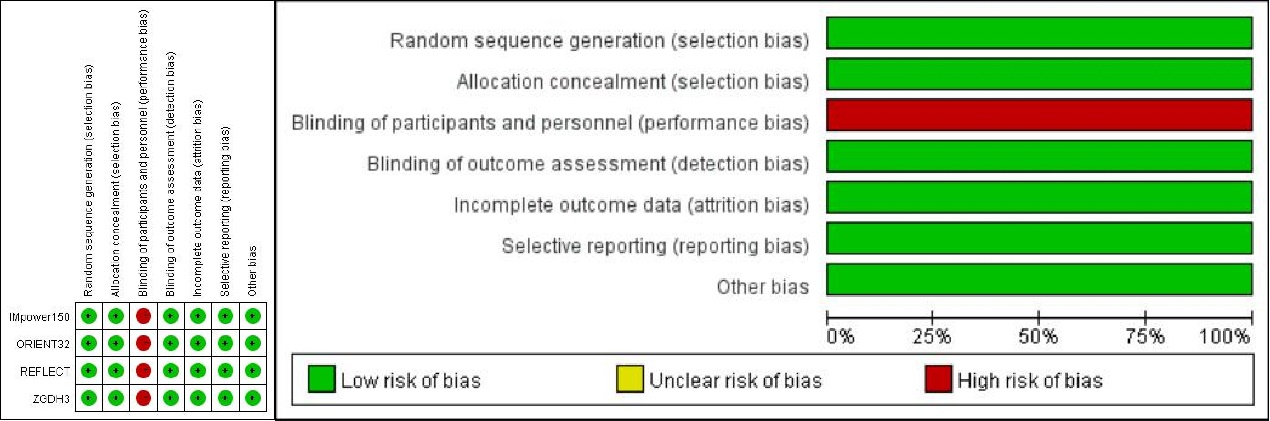


# eFigure 2 Summary of Results from Assess ment of Studies Using the Cochrane Risk of Bias Tool

**OS**

| **A** | **B** |
| --- | --- |
| **C** | **D** |

|  |  |
| --- | --- |
| **A** | **B** |
| **C** | **D** |

# eFigure 3 Log Cumulative Hazards Ratios Plots for OS and PFS

**PFS**

# (D+A, sintilimab plus bevacizumab; T+A, atezolizumab plus bevacizumab)

**OS**

**PFS**

# eFigure 4 OS and PFS Curves for the Best Fitted Fractional Polynomial Models

# (D+A, sintilimab plus bevacizumab; T+A, atezolizumab plus bevacizumab)

# eTbale 2 The Goodness-of-Fit Results for Fractional Polynomials models

| power | DIC | power | DIC |
| --- | --- | --- | --- |
| -2^a^ | 845.2 | -2^a^ | 1063.92 |
| -1 | 857.6 | -1 | 1079.76 |
| -0.5 | 870.3 | -0.5 | 1079.62 |
| 0 | 882.8 | 0 | 1072.42 |
| 0.5 | 890.7 | 0.5 | 1064.32 |
| 1 | 891.6 | 1^b^ | 1063.19 |
| 2 | 10562.0 | 2 | 1227.15 |
| 3 | 45170.8 | 3 | 170995 |

a: the smallest DIC among all chosen models; b: eliminated due to inconsistency with clinical reality

**
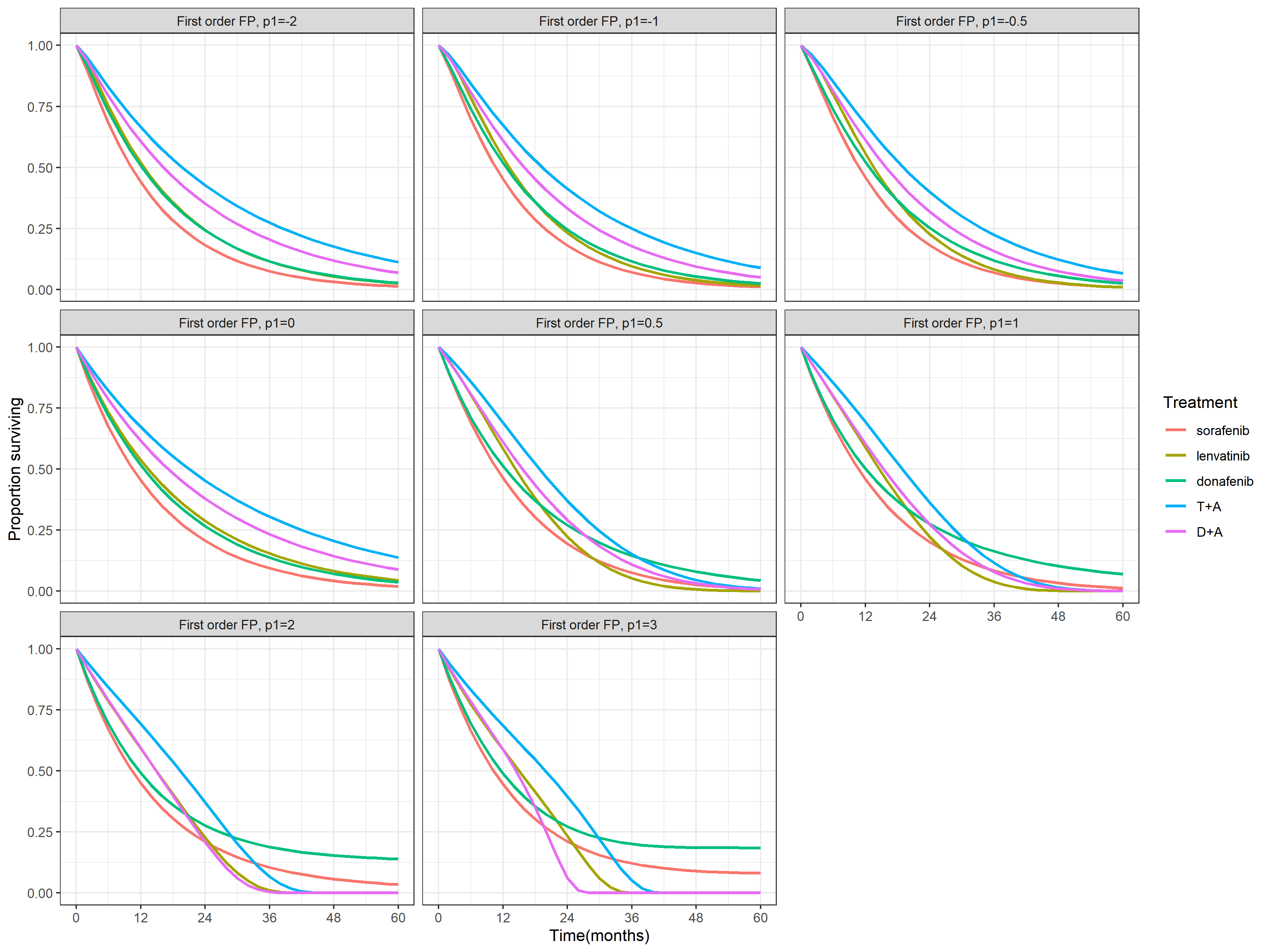
**

**OS**

**
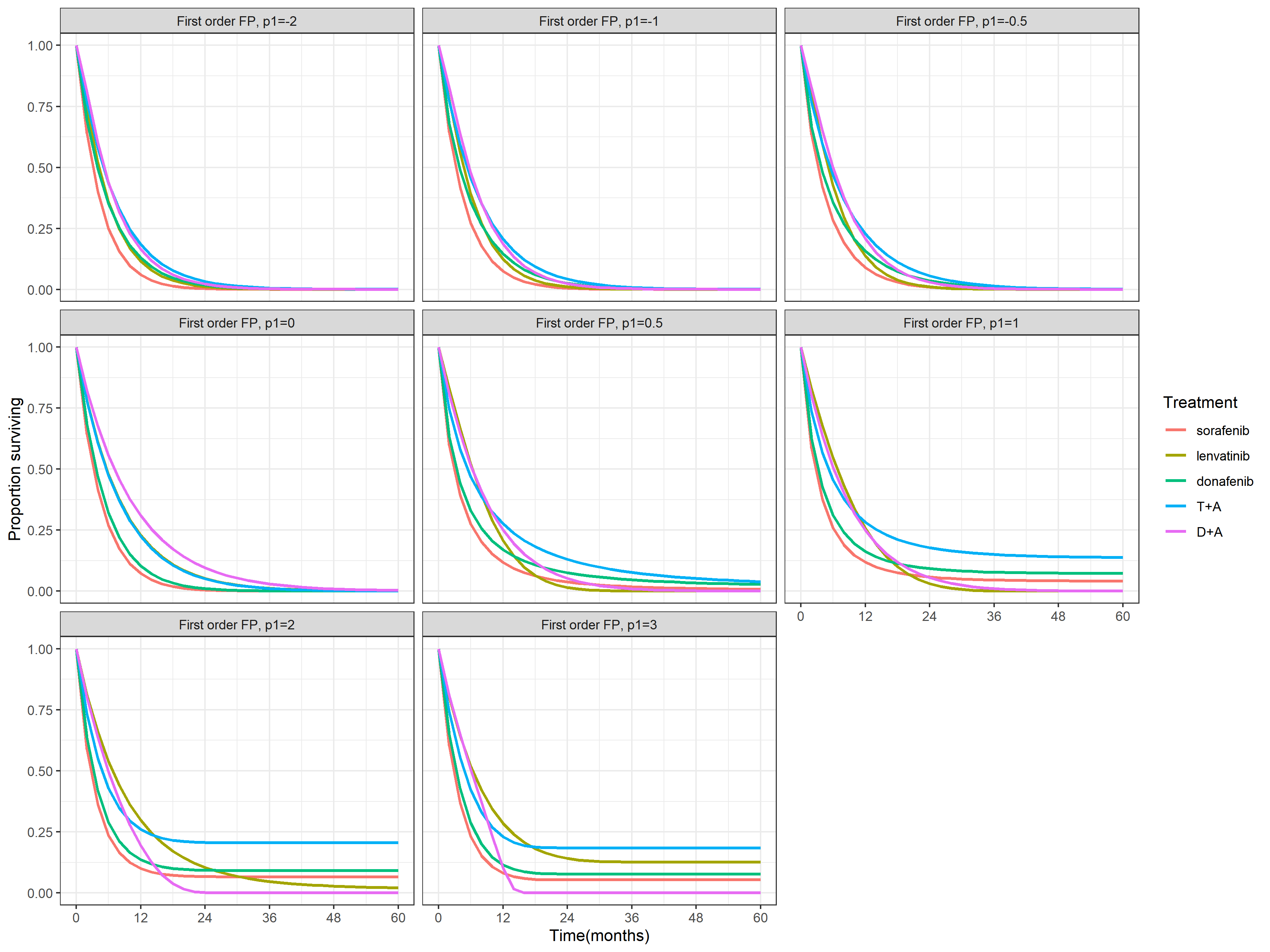
**

**PFS**

**eFigure 5** **OS and PFS Curves Fitted by All First-order Fractional Polynomial Models**

# eTable 3 The Goodness-of-Fit Results for Different Disturbutions for Sorafenib Curves

| OS | | | | | | |
| --- | --- | --- | --- | --- | --- | --- |
| disturbution | exponential | gamma | gompertz | Weibull | log-logsitic | log-normal |
| AIC | 1981.423 | 1954.837 | 1979.761 | 1962.437 | 1941.589 | 1942.024 |
| BIC | 1985.225 | 1962.441 | 1987.365 | 1970.365 | 1949.193 | 1949.628 |
| PFS | | | | | | |
| AIC | 1433.28 | 1396.791 | 1430.472 | 1421.44 | 1305.486 | 1308.666 |
| BIC | 1437.082 | 1404.395 | 1438.076 | 1429.044 | 1313.09 | 1316.27 |

# eTable 4 Adverse Reactions of Each Drug

| Grades 3 to 4 AE | Rate | | | | |  | Supposed duration | Drugs used | Cost for AE per duration ($) |
| --- | --- | --- | --- | --- | --- | --- | --- | --- | --- |
|  | Sorafenib | T+A | lenvatinib | D+A | Regorafenib | donafenib |  |  |  |
| Hypertension | 12.01% | 15.20% | 23.32% | 14.47% | 15.24% | 9.00% | 5 | Amlodipine and irbesartan tablets | 1.35 |
| Aspartate aminotransferase increase | 6.24% | 7.00% | 5.04% | 1.32% | 10.96% | 2.00% | 10 | Magnesium isoglycyrrhizinate injection | 87.30 |
| Diarrhoea | 4.04% | 1.80% | 4.20% | 1.58% | 3.21% | 2.00% | 5 | Loperamide hydrochloride capsules | 3.28 |
| Alanine aminotransferase increase | 0.86% | 3.60% | 0.00% | 1.32% | 3.21% | 2.00% | 10 | Magnesium isoglycyrrhizinate injection | 87.30 |
| Blood bilirubin increase | 5.02% | 2.40% | 7.77% | 5.00% | 10.43% | 1.00% | 8 | Ademetionine succinate for injection | 113.53 |
| Rash | 0.99% | 0.00% | 0.00% | 0.26% | 12.57% | 1.00% | 3 | Loratadine tablets | 2.60 |
| Platelet count decrease | 2.82% | 3.30% | 5.46% | 8.16% | 3.74% | 4.00% | 7 | Recombinant human thrombopoietin injection | 1054.22 |
| Palmar-plantar erythrodysesthesia syndrome | 10.90% | 0.00% | 2.94% | 0.00% | 0.00% | 3.26% | 14 | Eucerin cream | 33.51 |
| Total cost ($) | 45.63 | 47.03 | 96.49 | 94.23 | 64.31 | 48.10 | NA | NA | NA |

| Abbreviations: D+A, sintilimab plus bevacizumab; T+A, atezolizumab plus bevacizumab. |
| --- |

| OS |  |
| --- | --- |
| PFS | eFigure 6 OS and PFS Curves Fitted by All Distributions(KM, Kaplan-Meier) |

A

B

WTP=3 GDP

# eFigure 7 Probabilistic Sensitivity Analysis for Scenario Analysis: Scatter Plot (A) and Cost-Effectiveness Acceptability Curve (B, 10,000 iterations)

# (D+A, sintilimab plus bevacizumab; T+A, atezolizumab plus bevacizumab)

# eMethod

Using data of the IMbrave 150 trial Chinese subgroup, the T+A and sorafenib second-line treatment methods were obtained. As no related evidence was available for other regimens, according to the mechanism of action, we assumed that the second-line treatment regimen D+A was consistent with that of T+A and the second-line treatment regimens lenvatinib and donafenib were consistent with that of sorafenib. Since the ORIENT-32 trial reported that patients who progressed would no longer use the original drug, we assumed that this proportion of patients would be treated with tyrosine kinase inhibitor (TKI) treatment. For PD-(L)1 drugs, the longest medication time is 2 years, and patients in the PFS status after 2 years also receive TKIs ^[1]^. TKIs mainly include regorafenib, cabozantinib, and ramucirumab. Regorafenib is currently the only drug approved in China and real-world studies have shown that its efficacy is not affected by front-line immunotherapy ^[2]^, so regorafenib was chosen as the TKI drug. Tislelizumab and camrelizumab are currently the main immune drugs approved for the second-line treatment of liver cancer in China. The RATIONALE 301^[3]^ showed that the objective response rate (ORR) of tislelizumab in second-line or later-line treatment of uHCC is as high as 18.8%, and the incidence of adverse reactions grade 3 or above for tislelizumab is far lower than those other immunosuppressive agents. Additionally, tislelizumab is the most widely used drug clinically in second-line treatment, so it was selected as the second-line immunotherapy regimen. Best support care (BSC) refers to symptomatic drugs when patients experience pain, discomfort, etc.

**References**

1. Wen F, Zheng H, Zhang P, Liao W, Zhou K, Li Q. Atezolizumab and bevacizumab combination compared with sorafenib as the first-line systemic treatment for patients with unresectable hepatocellular carcinoma: A cost-effectiveness analysis in China and the United states. Liver Int. 2021 May;41(5):1097-1104. doi: 10.1111/liv.14795.
2. Yoo C, Byeon S, Bang Y, et al. Regorafenib in previously treated advanced hepatocellular carcinoma: Impact of prior immunotherapy and adverse events. Liver Int. 2020; 40: 22634– 2271.doi: 10.1111/liv.14496.
3. Qin S, Finn RS, Kudo M, et al. RATIONALE 301 study: tislelizumab versus sorafenib as first-line treatment for unresectable hepatocellular carcinoma. Future Oncol. 2019 Jun;15(16):1811-1822. doi: 10.2217/fon-2019-0097. Epub 2019 Apr 10. PMID: 30969136. doi: 10.2217/fon-2019-0097.
